# A machine learning tool for predicting newly diagnosed osteoporosis in primary healthcare in the Stockholm Region

**DOI:** 10.1038/s41598-025-24450-5

**Published:** 2025-10-20

**Authors:** Per Wändell, Axel C. Carlsson, Per Swärd, Julia Eriksson, Johan Ärnlöv, Andreas Rosenblad, Caroline Wachtler, Toralph Ruge

**Affiliations:** 1 Department of Neurobiology, Care Sciences and Society, Division of Family Medicine and Primary Care, NVS Department, Karolinska Institutet, Alfred Nobels Allé 23, 141 83 Solna, Huddinge, Sweden; 2 Academic Primary Health Care Centre, Region Stockholm, Stockholm, Sweden; 3 Clinical and Molecular Osteoporosis Research Unit, Departments of Orthopedics and Clinical Sciences, Skåne University Hospital, Lund University, Malmö, Sweden; 4 Division of Biostatistics, Institute of Environmental Medicine, Karolinska Institutet, Stockholm, Sweden; 5 School of Health and Social Studies, Dalarna University, Falun, Sweden; 6 Regional Cancer Centre Stockholm-Gotland, Region Stockholm, Stockholm, Sweden; 7 Department of Medical Sciences, Division of Clinical Diabetology and Metabolism, Uppsala University, Uppsala, Sweden; 8 Department of Emergency and Internal Medicine, Skånes University Hospital, Malmö, Sweden; 9 Department of Clinical Sciences Malmö, Department of Internal Medicine, Lund University, Skåne, Sweden

**Keywords:** Osteoporosis, Primary care, Machine learning, Vertebral fractures, Epidemiology, Outcomes research, Metabolic bone disease

## Abstract

Improving accuracy and timeliness for osteoporosis diagnosis could help prevent fragility fractures, morbidity, and mortality for older individuals. Osteoporosis is an often silent health condition, especially as regards vertebral fractures, and WHO issued a call to action for primary care to lead efforts in screening, assessing, and managing diseases such as osteoporosis. We used a machine learning method, Stochastic Gradient Boosting (SGB), to identify what diagnoses in a primary care setting predict a new osteoporosis diagnosis, using a sex- and age-matched case–control design. Cases of new osteoporosis (ICD-10 code: M80, M81, M82) were identified across all outpatient care settings during 2012–2019. We included individuals aged ≥ 40 years old, stratified by sex and age-groups 40–65 years and > 65 years old. Controls were sampled from outpatients that did not have osteoporosis at any time during 2010–2019. Using the SGB model, we ranked the most important diagnoses related to newly diagnosed osteoporosis, presented as the normalized relative influence (NRI) score with a corresponding odds ratio of marginal effects (OR_ME_) of being newly diagnosed with osteoporosis. A train-test approach was used to develop the model, with the performance evaluated using area under the curve (AUC). In total**,** we included 30,741 patients with osteoporosis aged ≥ 40 years. AUC was high, > 0.899 for all age and sex stratas. The number of visits to primary care in the year prior to the osteoporosis diagnosis contributed with the most predictive information for all age and sex stratas. For all age groups several other factors also showed high NRI and OR_ME_ and among them many unspecific diagnoses such as Dorsalgia showed high NRI, (2.6–9.0%) and other painful musculoskeletal disorders. However, our study also showed that the diagnosis of Hypertension had a very high NRI for patients aged > 65 years but not in patients 40–65 years of age. In this AI study, including only diagnoses from patients seen in primary health care centres, we found that the number of consultations in primary care had high predictive information as well unspecific diagnoses including muscle and skeletal pain predicted high risk for osteoporosis in all age groups.

## Introduction

Osteoporosis is an often silent health condition and an increasing challenge for health care and society because of the world’s ageing population^1^. Osteoporosis is a common metabolic bone disorder with a strong hereditary influence whereas as much as 75% of the variability in bone mineral density (BMD) can be explained by heritability^2^. Genetic markers only explain about 20% of the variability in BMD, according to a study from the UK biobank of more than 400,000 individuals^3^. Osteoporosis development is influenced by lifestyle habits such as diet and physical activity throughout life^4^. It is characterized by low bone mass, structural deterioration of bone tissue, and disruption of bone microarchitecture, as well as risk of fractures after mild or minimal trauma, i.e., osteoporotic or fragility fractures. Osteoporosis diagnosis is primarily based on BMD as assessed by dual-energy X-ray absorptiometry (DXA)^5^. Fractures of the hip, forearm, humerus, pelvis, and vertebrae are most often attributable to osteoporosis^6,7^. Individuals with osteoporosis have a higher risk of all-cause mortality and increased healthcare costs^8^, especially in developed countries^9^, including the EU^10^. Geographic and ethnic patterns differ globally^11,12^. Fragility fractures are especially common in Northern Europe. In a Norwegian study, the incidence of hip and forearm fractures were shown to be higher than in studies from other countries^13^. Fractures place a severe burden on aging individuals since they may lead to poor quality of life and increased mortality^14^. The World Health Organization (WHO) defined osteoporosis as BMD being 2.5 SD or more below the average value for young healthy women (*t*-score <  − 2.5 SD). A second threshold describes “low bone mass” or osteopenia as a *t*-score that lies between − 2.5 and − 1.0 SDs. WHO issued a call to action for primary care to lead efforts in screening, assessing, and managing non-communicable diseases, including osteoporosis^15^.

Osteoporosis remains underdiagnosed and undertreated and suspected osteoporosis is often only investigated once the individual has a low energy fracture^16^. The main risk factors for fragility fractures and altered bone metabolism are well known and include older age, female sex, ethnicity, heredity, previous fracture, malnutrition, alcohol overconsumption, current smoking, vitamin D deficiency, physical inactivity, various medications, and medical disorders^17,18^. Diagnosis of osteoporosis has important implications for patient care. Vertebral fractures, one of the most common osteoporotic fractures, is often underdiagnosed and greatly influences patient mobility and quality of life^19,20^. Hip fractures are of high interest as they affect individual’s function profoundly and require hospitalization and surgery^21^. Current knowledge about risk factors for osteoporosis is not applied systematically. Fracture risk can be assessed by the Fracture Risk Assessment Tool (FRAX®)^5^. However, FRAX is not ideal in all circumstances^22–24^, and addition of Trabecular Bone Score (TBS) assessment to FRAX and/or BMD enhances fracture risk prediction^25^. Besides, different fall risk indices are used clinically^26^, and the Downton Fall Risk Index has been shown to predict hip fractures^27^. Even emergent fractures do not always lead to further investigations into bone mass status^16,28^. A systematic approach to identify of osteoporosis risk based on automatic analysis of routinely collected clinical information could improve osteoporosis diagnosis frequency leading to reduced fracture risk.

With the help of machine learning (ML) and artificial intelligence (AI) large amounts of clinical datasets can be assessed and prediction of diseases based on underlying variables and patterns can be understood. Different ML and AI models have been developed in order to predict osteoporosis considering traditional factors known to be associated with increased risk of osteoporosis. In brief, four main areas have been approached, bone properties assessment, osteoporosis classification, fracture detection, and risk prediction^29^. Yoo et al. and Kim et al. developed ML models with the aim of identifying risk of osteoporosis in Korean postmenopausal women, a group with a particular increased risk for osteoporosis^30^. Critical variables such as age, height, weight, body mass index, duration of menopause, duration of breast feeding, estrogen therapy, hyperlipidemia, hypertension, osteoarthritis, and diabetes mellitus were identified. Suh et al. constructed a deep-learning model with clinical risk factors for osteoporosis with an AUC 0.85–0.92 identifying sex, age, BMI as well as chronic diseases as important variables^31^. Patients with chronic diseases have an increased risk for osteoporosis and ML models have been developed to predict osteoporosis in patients with rheumatoid arthritis^32^, identifying risk factors such as Body mass index, age, menopause, waist and hip circumferences, RA surgery, and monthly income) as well as in patients with type 2 diabetes^33^, and in postmenopausal women with diabetes^34^.

In summary, early identification and treatment of osteoporosis could lead to significant benefits for patients, healthcare, and society. We believe that the use of ML and AI has a great potential by helping the clinicians with predictive models. In contrast to previous performed ML models, our research approach was to examine the feasibility of utilizing primary care diagnoses and healthcare-seeking behaviours to identify individuals with elevated risks of osteoporosis. We hypothesized that discernible diagnostic patterns and healthcare-seeking behaviours may reveal patterns preceding a formal diagnosis of osteoporosis, which may increase the awareness of osteoporosis and leveraged to improve identification and treatment of in primary care.

## Methods and material

### Study design and participants

This study used a sex- and age-matched case–control design that sought to develop a predictive model for osteoporosis using a machine learning algorithm. The controls were selected from individuals who had not received a diagnosis of osteoporosis within our study period (2010–2019). Up to ten controls were individually matched to each case, resulting in a total of 338,151 individuals included in the study. The study used an AI-method, Stochastic Gradient Boosting (SGB), to identify relevant diagnoses for detecting new osteoporosis in a primary care setting^35^. The study population consisted of individuals aged ≥ 40 years registered at primary health care centres (PHCCs) in Region Stockholm, an area with a population of approximately 2.4 million residents. Data for this study were gathered from the VAL-databases^36^, which encompass all registered diagnoses based on the International Classification of Diseases, 10th Revision (ICD-10). Apart from very few private clinics that operate without subsidies in Stockholm, all appointments and diagnoses are recorded and stored in VAL, the central regional data warehouse. The data for this study were collected specifically from visits to PHCCs in the Stockholm Region. It has been estimated that approximately 95% of all physician appointments in primary care have at least one diagnosis recorded and stored in VAL. Cases of incident osteoporosis (ICD-10 codes: M80, M81, M82) were identified across all outpatient care settings 2012–2019 (without an osteoporosis diagnosis in 2010–2011), but data on the diagnoses and health care utilization used for predicting new osteoporosis diagnosis were exclusively obtained from the PHCCs, to mimic what is available in patient records in primary care. The controls were sampled from the outpatients in 2012–2019 that did not have osteoporosis at any time during 2010–2019.

Cases and controls were identified based on medical record data from the years 2012–2019. For controls, we examined diagnoses registered during the three years prior to the diagnosis date of the matched case (index date). We identified 31,580 individuals with a new osteoporosis diagnosis during 2012–2019 (who had no osteoporosis diagnosis registered during 2010–2011). The present study’s focus on patients ≥ 40 years old was due to osteoporosis being extremely rare in younger individuals. Thus, we included 30,741 patients ≥ 40 years old with osteoporosis.

### Statistical analyses

The SGB technique is a form of machine learning utilized in related research^37^. For instance, tree-based machine learning methods such as SGB have been recommended in a recent meta-analysis of machine learning tools for detecting diabetes^38^, and we have recently published an article on the detection of new diabetes^39^. SGB models that were applied used Bernoulli loss function with up to 20,000 trees, and a depth of maximum 5 interactions. The shrinkage (learning rate) was set to of 0.01, and minimum 10 observations in the terminal nodes. The subsampling rate (bag fraction) was 0.5 and a10-fold cross-validation was used to obtain the optimal number of trees.

We listed all diagnoses during the three years before the index date for all individuals based on a list of the top 2000 most common diagnoses registered in primary care^40^.

As risk factors for osteoporosis are divergent in men and women, and the diagnosis pattern is different in men and women, we performed sex-stratified analyses. We split the entire dataset into males and females, as well as in younger (40–65 years old) and older adults (> 65 years old). For each group, we split the data into a 50% training set and a 50% test set, ensuring that the proportion of individuals with osteoporosis was roughly equal between the training and test data sets.

For each training data set, we selected diagnoses with at least 10 occurrences, resulting in 177 diagnoses for men aged 40–65, 174 diagnoses for men aged > 65, 252 diagnoses for women aged 40–65 and 320 diagnoses for women aged > 65. The performances of the final models were evaluated using area under the receiver operator characteristics (ROC) curve (AUC), sensitivity, and specificity. The SGB model was then applied to each test data set to obtain patient-specific probabilities of being newly diagnosed with osteoporosis. The probabilities that maximized the sum of sensitivity and specificity were used as cut-off values, so that patients with a probability higher than this cut-off were classified as being newly diagnosed with osteoporosis. The results are presented in a confusion matrix with performance of the prediction given by sensitivity and specificity.

From the SGB model, we ranked the most important diagnoses related to newly diagnosed osteoporosis, presented as the normalized relative influence (NRI) score with a corresponding odds ratio of marginal effects (OR_ME_) of being newly diagnosed with osteoporosis. For each diagnosis, the odds ratio was calculated using the probabilities of being newly diagnosed with osteoporosis obtained by integrating out all other variables in the model using the weighted tree traversal method. The analyses were performed using R version 4.2.1^41^, with the R package ‘gbm’ used for the SGB analyses.

### Ethics

The study plan was approved by the Swedish Ethical Review Authority and all data were pseudonymized to protect patient privacy. All methods were carried out in accordance with relevant guidelines and regulations, and as with all register based studies without recruitment of participants by invitation, a waiver of informed consent was granted. The data in the present study are available for research purposes by qualified researchers trained in human subject confidentiality protocols after ethical approval from Region Stockholm at halsodata.rst@regionstockholm.se.

## Results

**Fig. 1:**
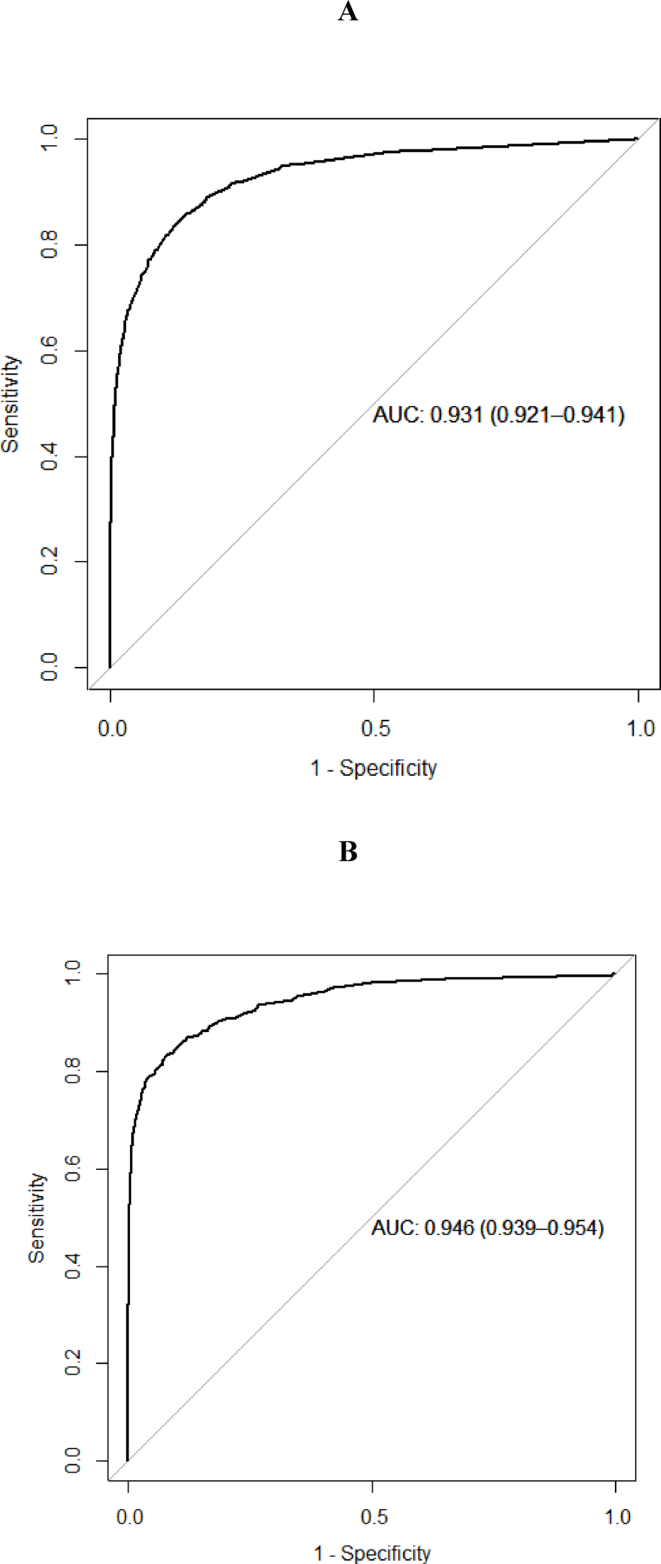
(**A**, **B**) Receiver operator characteristics curve for the optimal stochastic gradient boosting model applied to the men in the test data set. **A** denotes men 40–65 years, and **B** men > 65 years.

**Fig. 2:**
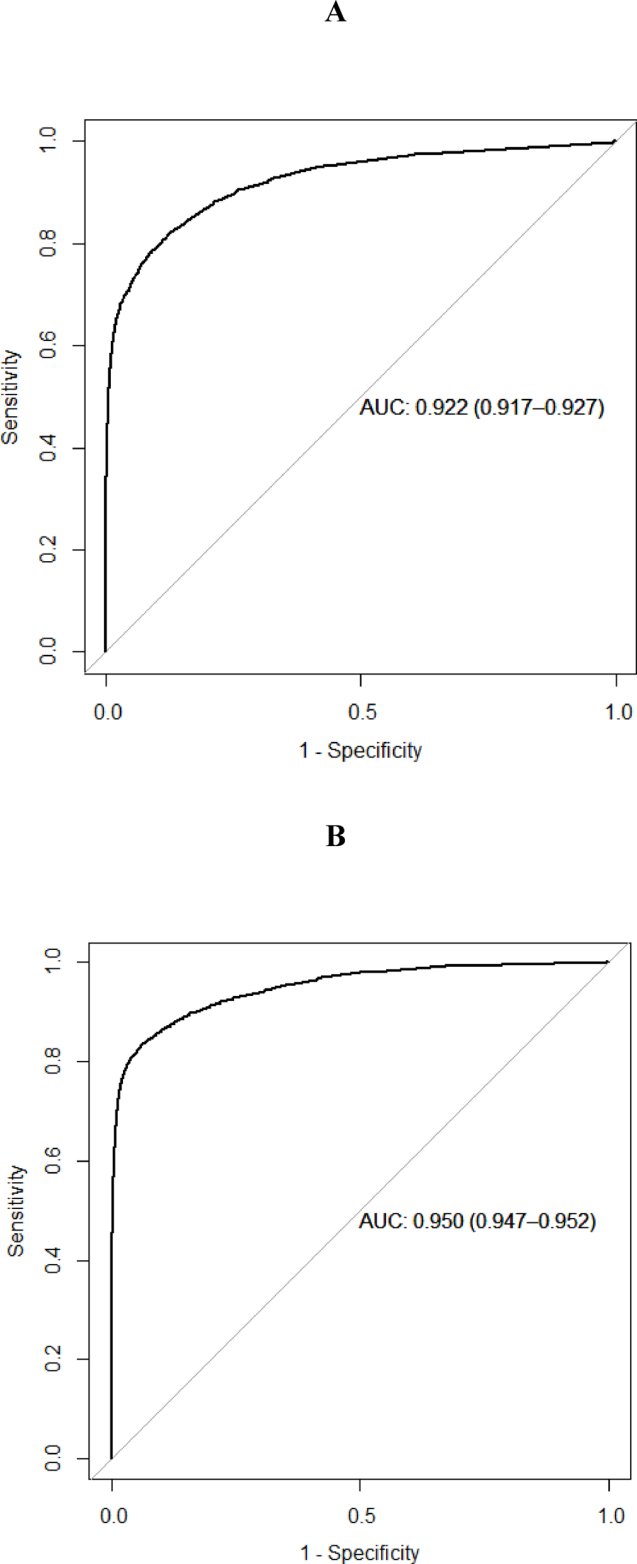
(**A**, **B**) Receiver operator characteristics curve for the optimal stochastic gradient boosting model applied to the women in the test data set. **A** denotes women 40–65 years, and **B** women > 65 years.

We identified 30,741 patients ≥ 40 years of age with osteoporosis. Since controls were sex- and age-matched, there were no significant age differences between cases and controls. In summary, the model showed an AUC of 0.931 for men 40–65 years and 0.946 for men > 65 years old, and for women an AUC of 0.922 for women 40–65 years and 0.950 for women > 65 years old (Figs. 1 and 2).

Among the 46,622 included men, 3974 (8.5%) had an osteoporosis diagnosis. For men aged 40–65 years, 8158 were included in the training group where 792 (9.7%) had an osteoporosis diagnosis, and 8158 were included in the test group where 786 (9.6%) had osteoporosis. Among men aged > 65 years, 15,153 were included in the training group where 1189 (7.8%) had osteoporosis, and 15,153 were included in the test group where 1207 (8.0%) had osteoporosis.

Among women, 291,529 were included where 26,767 (9.2%) had an osteoporosis diagnosis. For women aged 40–65 years, 4330 (9.3%) of the 46,518 in the training group had osteoporosis, while 4343 (9.3%) of the 46,517 included in the test group had osteoporosis. For women aged > 65 years, 99,247 were included in the training group where 8865 (8.9%) had osteoporosis, and 99,247 were included in the test group where 9229 (9.3%) had osteoporosis.

### Ability of prediction of the SGB model

The predictive ability of the SGB model is presented in Table 1, which shows the confusion matrix for men and women based on diagnoses made 3 years before the osteoporosis diagnosis in the test dataset. The SGB model used a maximum of 20,000 decision trees; for men 40–65 years 1023 trees, with sensitivity: 0.837, and specificity 0.888; for men > 65 years 2617 trees, with sensitivity 0.833, and specificity 0.920; for women 40–65 years 8208 trees, with sensitivity 0.822, and specificity 0.873; and finally for women > 65 years 12,582 trees, with sensitivity 0.837, and specificity 0.936.

**Table 1:** Confusion matrix for predicting presence of new osteoporosis among men and women in the test dataset using the optimal number of stochastic gradient boosting trees.

Predicted	Observed		
	Not osteoporosis	Osteoporosis	Total
Men 40–65 years			
Not osteoporosis	6484	128	6612
Osteoporosis	888	658	1546
Total	7372	786	8158
Men > 65 years			
Not osteoporosis	12,833	201	13,034
Osteoporosis	1113	1006	2119
Total	13,946	1207	15,153
Women 40–65 years			
Not osteoporosis	36,822	772	37,594
Osteoporosis	5352	3571	8923
Total	42,174	4343	46,517
Women > 65 years			
Not osteoporosis	84,293	1508	85,801
Osteoporosis	5725	7721	13,446
Total	90,018	9229	99,247

### Variable importance

Table 2 presents the 10 diagnoses with the highest NRI specifically for men, while Table 3 does the same for women. For men aged 40–65 years, the top five factors with the highest relative influence (NRI) were “Number of visits to primary care 12 months before diagnosis”, followed by “Hypertension”, “Encounter for medical observation for suspected diseases and conditions ruled out”, "Dorsalgia”, and “Persons encountering health services in other circumstances”. For men aged > 65 years, the top five diagnoses with the highest relative influence (NRI) were “Number of visits to primary care 12 months before diagnosis”, “Hypertension”, "Dorsalgia”, “Encounter for other special examination without complaint, suspected or reported diagnosis”, and “Other and unspecified soft tissue disorders, not elsewhere classified”.

**Table 2:** Results of the predicted presence of new osteoporosis diagnosis among men using the optimal stochastic gradient boosting (SGB) model with 20,000 trees, together with odds ratios for marginal effects (OR_ME_) of osteoporosis.

ICD-10 code	Description	NRI (%)	OR_ME_
40–65 years			
Visits	Number of visits 12 months before diagnosis	72.2	6.5
Hypertension	I10, I11, I12, I13, I15	3.2	1.7
Z03	Encounter for medical observation for suspected diseases and conditions ruled out	3.1	1.5
M54	Dorsalgia	2.6	1.8
Z76	Persons encountering health services in other circumstances	2.4	1.7
M25	Other joint disorder, not elsewhere classified	1.8	2.2
J06	Acute upper respiratory infections	1.7	2.2
Painful condition	K58, R52, B02, G50, G35, G53, G54, G55, G56, G57, G58, G60, G61, G62, G63, G95	1.1	1.9
M79	Other and unspecified soft tissue disorders, not elsewhere classified	0.9	1.4
Asthma	J45, J46	0.8	7.8
> 65 years			
Visits	Number of visits 12 months before diagnosis	31.8	9.2
Hypertension	I10, I11, I12, I13, I15	20.2	5.4
M54	Dorsalgia	9.0	14.7
Z03	Encounter for medical observation for suspected diseases and conditions ruled out	2.9	2.3
M79	Other and unspecified soft tissue disorders, not elsewhere classified	2.8	4.1
I48	Atrial fibrillation and flutter	1.9	2.0
Painful condition	K58, R52, B02, G50, G35, G53, G54, G55, G56, G57, G58, G60, G61, G62, G63, G95	1.9	1.9
M48	Other spondylopathies	1.8	15.2
Z76	Persons encountering health services in other circumstances	1.8	0.7
Z92	Personal history of medical treatment	1.5	3.0

The 10 variables for men with highest normalized relative influence (NRI) are shown.

**Table 3:** Results of the predicted presence of new osteoporosis diagnosis among women using the optimal stochastic gradient boosting (SGB) model with 20,000 trees, together with odds ratios for marginal effects (OR_ME_) of osteoporosis.

ICD-10 code	Description	NRI (%)	OR_ME_
40–65 years			
Visits	Number of visits 12 months before diagnosis	43.6	10.4
M54	Dorsalgia	5.3	3.8
Z03	Encounter for medical observation for suspected diseases and conditions ruled out	5.0	2.2
M79	Other and unspecified soft tissue disorders, not elsewhere classified	5.0	3.0
Hypertension	I10, I11, I12, I13, I15	4.0	1.8
Painful condition	K58, R52, B02, G50, G35, G53, G54, G55, G56, G57, G58, G60, G61, G62, G63, G95	3.4	2.5
Z76	Persons encountering health services in other circumstances	2.8	1.6
J06	Acute upper respiratory infections	2.7	2.6
M25	Other joint disorder, not elsewhere classified	2.3	2.0
Thyroid disorders	E02, E03, E07	1.7	5.3
> 65 years			
Hypertension	I10, I11, I12, I13, I15	27.1	11.7
Visits	Number of visits 12 months before diagnosis	26.7	12.0
M54	Dorsalgia	6.4	9.4
Z03	Encounter for medical observation for suspected diseases and conditions ruled out	5.4	2.8
M79	Other and unspecified soft tissue disorders, not elsewhere classified	4.3	4.6
Painful condition	K58, R52, B02, G50, G35, G53, G54, G55, G56, G57, G58, G60, G61, G62, G63, G95	3.5	4.1
Z76	Persons encountering health services in other circumstances	3.4	1.4
Thyroid disorders	E02, E03, E07	1.7	4.5
Chronic obstructive pulmonary disease	J41, J42, J43, J44	1.1	5.3
Z00	General medical examination	1.0	1.7

The 10 variables for women with highest normalized relative influence (NRI) are shown.

Similarly, for women aged 40–65 years (Table 3), the top five diagnoses with the highest NRI were “Number of visits 12 months before diagnosis”, Dorsalgia, “Encounter for other special examination without complaint, suspected or reported diagnosis”, “Other and unspecified soft tissue disorders, not elsewhere classified”, and “Hypertension”; and for women > 65 years “Hypertension”, “Number of visits to primary care 12 months before diagnosis”, “Dorsalgia”, “Encounter for other special examination without complaint, suspected or reported diagnosis”, and “Painful condition”.

For marginal effects (Tables 2 and 3), the results for the sex and age-group stratified models showed that the top 5 diagnoses with the highest NRI all had an OR_ME_ > 1.

## Discussion

In this study when using a machine learning algorithm to detect osteoporosis, we found different contributing factors. For instance, the number of visits to primary care in the year prior to the osteoporosis diagnosis contributed with the most predictive information, compared to specific diagnoses that the patient received at these multiple visits. In addition, diagnosis of painful conditions, visits for unspecific symptoms, and diagnosis of hypertension all contributed to prediction of an osteoporosis diagnosis for both sexes in all age categories.

Recent findings underline that different screening approaches may be needed to identify patients at risk of fragility fracture. The Risk-stratified Osteoporosis Strategy Evaluation (ROSE) randomized trial identified some shortcomings in the algorithm; FRAX to select women for DXA scan followed by (in women with DXA verified osteoporosis) a recommendation to discuss anti-osteoporotic medication with their GP. Overall gains regarding fracture risk and mortality were only observed in women at moderate to high risk who went on to undergo DXA scans. Women who declined or did not attend an offered DXA (29%) were characterized by higher age, living alone and higher osteoporosis risk profile^24^. It was suggested, that combining traditional screening methods with automated digital detection tools including the Fracture Risk Evaluation Model (FREM) or the Fracture Liaison Service (FLS)^42^, could lead to the identification of a larger population at risk^24^.

### Results in perspective

Several attempts to predict the risk for osteoporosis with the help of AI and machine learning have been done, with subsequent reviews and meta-analyses being performed^29,43–45^. Risk prediction models have included the risks of bone density loss (BMD), fragility fractures, falls, or comorbidities^29^; BMI, pregnancy, duration of menopause, estrogen therapy, or co-morbidities such as osteoarthritis, rheumatoid arthritis, or diabetes^43^. Compared to these models we identified diagnoses and factors from the primary care medical records, i.e. we used another approach. It is difficult to compare our results with results from the different included studies owing to the great variety of methods.

Most low energy hip and radius fractures are detected and treated at emergency departments at hospitals, but many vertebral fractures are asymptomatic or rather misdiagnosed. A US study showed that more than two thirds of vertebral fractures are undetected^46^. Vertebral fracture is a common fragility fracture^47,48^, and is prognostic for a higher risk of future vertebral fractures^49^ as well as for other fractures, especially hip fractures^50^.

The definition of osteoporosis in this study depends on the clinician setting the diagnosis. In this study, cases are defined as having an osteoporosis diagnosis with or without fracture, and we lack information whether the cases have a DXA preceding diagnosis or not. However, fragility fractures are among the most obvious clinical manifestations of osteoporosis which could lead to treatment and diagnosis without a previous DXA, particularly in elderly patients, why it could be presumed that the cases identified are likely to represent true osteoporosis^51^. However, vertebral fractures are to a large rate undetected^46^, why this is a crucial point for detecting osteoporosis early and initiate treatment.

In this study, search patterns prior to osteoporosis diagnosis differed very little between men and women, and incidence of osteoporosis was higher in female patients. It is well accepted that women develop osteoporosis at a younger age due to the sudden drop in hormone (estrogen) levels at menopause. Decrease in bone mass occurs at a slower rate in men than in women until the age of 65–70 years, when the rate is the same for men and women, and women tend to have fractures about 5–10 years earlier than men^52^. The most plausible explanation for our findings is that our data probably only represents patients seen in PHCCs. This means that they do not include patients presenting with fragility fractures at the emergency department where the sex difference may be more pronounced.

In this study, the diagnosis dorsalgia was highly predictive of osteoporosis, especially among women, but there were also other pain related diagnoses such as unspecific painful conditions, joint pain, or unspecified pain, which had high predictive values for osteoporosis in both men and women. “Dorsalgia” is an unspecific diagnosis in primary health care and one of the most common diagnoses in Sweden, accounting for 2.6% of all primary health diagnoses^40^. It is possible that the “dorsalgia” diagnosis could reflect a fragility vertebral fracture, especially in the case of recent trauma, prolonged use of corticosteroids, age, structural spinal deformity, and loss of height^46,50^. In a Japanese study among women aged > 70 years old with acute lower back pain, as many as 77% had a vertebral fracture, with occult fractures amounting for 33% of the total number of vertebral fractures^53^. Our study suggests that unspecific pain in a patient may merit further investigation of potential vertebral fractures. It is known that vertebral fractures (the most common fragility fracture) can occur non-traumatically, are vastly underdiagnosed, and often mistaken for unspecific skeletal and joint pain^54^. It was recently shown that the identification of an occult vertebral fracture predicted future fragility fractures, including hip fractures, independently of BMD and clinical risk factors from FRAX^55^. Our findings further reinforce that osteoporosis is underdiagnosed. A review of osteoporosis prevalence found a global rate of 18.3%, and a European rate of 18.6%^56^.

Finally, our study also identified patients with chronic diseases as a group of patients with an increased risk of being diagnosed with osteoporosis. This is in concert with the literature where previous data have established a well-accepted risk for osteoporosis in patients with chronic diseases. Interestingly, this group of patients stands out as a specific risk group in primary health care. Patients with chronic diseases have a high consumption of health care and are often seen as frequent visitors at different health care centres.

### Strengths and limitations

There are limitations as well as strengths to this study. We studied individuals who received a osteoporosis diagnosis, but individuals with as yet undetected osteoporosis might have other patterns. For example, undetected or false negative rates of vertebral fractures have been shown to be between 34%^57^ and 68%. Even though the model showed that certain diagnoses were predictive for a later osteoporosis diagnosis, it remains to be shown how this information can inform clinical choices about further investigation. Among the strengths is that we base this study on a database containing the total population of residents from Region Stockholm.

### Clinical implications

Because traditional prognostic models are always built on theoretical, hypothetical relationships between known risk factors and the outcome, AI methodology adds value—because it is explorative and can identify previously unknown relationships. However, the prognostic value of these factors needs to be externally validated before it can be used clinically. The current main use of the findings is the study contribution to the etiology of osteoporosis and that the findings of the study may be used as hypothesis generating as the predicting diagnoses may be viewed as risk factors for osteoporosis. We are collaborating with software developers with suitable platforms, where our algorithms may be tested and implemented in the future (https://www.blackwell.se/), that may be used by doctors, together with the patient record data to identify undiagnosed patients in primary care. This is, however, not yet in place.

## Conclusions

In conclusion, the utilization of machine learning for the identification of patients with osteoporosis in primary care is not merely a technological advancement but could be a strategic imperative, in finding patterns preceding a diagnosis, and thus underlining a more detailed examination. As the field of healthcare continues to evolve, embracing machine learning in the primary care setting could possibly be a useful step towards achieving precision medicine and addressing the growing public health challenge posed by osteoporosis.

## Data Availability

The data that support the findings of this study are available from Region Stockholm at halsodata.rst@regionstockholm.se, but restrictions apply to the availability of these data, which were used under license for the current study, and so are not publicly available. Data are however available from the authors upon reasonable request and with permission of Region Stockholm.

## Abbreviations

BMD: Bone mineral density
NRI: Normalized relative influence
PHCC: Primary health care centre
SD: Standard deviation
SGB: Stochastic gradient boosting
